# Teacher-related factors and students' academic performance: a multilevel analysis

**DOI:** 10.3389/fpsyg.2026.1839853

**Published:** 2026-08-04

**Authors:** Wusen Qiu, Yuekun Shao, Weixin Lin, Zehe Yin, Chaoqiao Yang, Yao Yao

**Affiliations:** 1 College of Design, Hainan Vocational University of Science and Technology, Haikou, China; 2 Chinese International College, Dhurakij Pundit University, Bangkok, Thailand

**Keywords:** academic performance, self-efficacy, teacher professional identity, teacher-student relationship, teaching innovation

## Abstract

**Background:**

This study examines the influence of teacher-related factors on students' academic performance in the post-COVID-19 context using a multilevel framework, drawing on Social Cognitive Theory and Self-Determination Theory.

**Methods:**

Data were collected from 40 teachers and 418 students across 10 universities in Hainan, China. Confirmatory factor analysis (CFA) and common method variance (CMV) tests were conducted. Hierarchical linear modeling (HLM) was employed to examine the cross-level relationships among teacher-student relationships, teaching innovation, teacher professional identity, student self-efficacy, and academic performance.

**Results:**

The results indicate that teacher-student relationships and teacher professional identity are significantly associated with students' academic performance, whereas teaching innovation shows no significant association. Furthermore, student self-efficacy positively predicts academic performance and mediates the relationship between teacher-student relationships and academic performance.

**Conclusion:**

These findings highlight the critical role of relational and psychological mechanisms in post-pandemic educational recovery.

## 1 Introduction

The COVID-19 pandemic in early 2020 shut down schools in many countries and profoundly impacted the education sector (Huber and Helm, 2020). More than 2,900 colleges in China have postponed or suspended classes, with many schools shifting to online learning (Leung and Sharma, 2020). As higher education globally and in China transitions into the post-pandemic era, these institutions continue to grapple with the long-term disruptions to traditional pedagogical structures, necessitating a deeper investigation into the factors that sustain educational quality (Aristovnik et al., 2020).

This shift has placed teachers under significant pressure to deal with new technologies and unfamiliar teaching methods, creating additional work requirements (Flack et al., 2020). Basilaia and Kvavadze (2020) found that online education was successful among students during the COVID-19 pandemic. However, students who underwent homeschooling had less support and feedback from teachers and received more written feedback—which provides fewer opportunities for guidance and help than verbal feedback—from their teachers (Mælan et al., 2021). Consequently, underachieving students learn less at home than in school (Bakken et al., 2020). These students tend to reduce their efforts and self-efficacy, which may lead to an even more significant gap between underachieving and well-performing students (Mælan et al., 2021).

Karakaya et al. (2020) found that teachers' concerns about shaping the post-pandemic social structure should be considered, including those pertaining to academic deficiencies, the loss of school atmosphere, and education itself. Cao et al. (2020) found that in China, the pandemic caused severe anxiety disorders among college students. A lack of support from teachers may lead to reduced self-efficacy during homeschooling, affecting the ability of underachieving students to maintain active learning (Klassen, 2010). However, Sintema (2020) found that the education provided during the COVID-19 pandemic negatively affected academic performance. Therefore, further developments of these methods are required. This may put more pressure on teachers, especially when they teach face-to-face (Kim and Asbury, 2020). These ongoing challenges underscore a critical area of concern: the need to further understand how teacher-level professional adaptations and student-level psychological resources might jointly interact to help restore academic success in post-disaster educational environments.

To systematically explain how these teacher and student factors may interact, this study integrates Social Cognitive Theory (SCT) and Self-Determination Theory (SDT). According to Bandura's (1977) Social Cognitive Theory, individuals‘ psychological capabilities, such as self-efficacy, are significantly shaped by environmental factors and observational learning. In the classroom context, teachers' instructional strategies can serve as critical environmental inputs that may enhance or inhibit students' cognitive beliefs (Klassen and Tze, 2014). Concurrently, Self-Determination Theory (Deci and Ryan, 2000) posits that individuals tend to exhibit optimal functioning and motivation when their basic psychological needs—particularly the need for relatedness—are satisfied by their social environment. Within this integrated framework, a supportive teacher–student relationship and a robust teacher professional identity are conceptualized as essential environmental and relational nutrients that help fulfill students' psychological needs, thereby potentially fostering their self-efficacy and, in turn, supporting their academic performance.

The COVID-19 pandemic has significantly reshaped higher education systems worldwide. While prior research has emphasized instructional strategies, limited attention has been given to the combined influence of teacher-level and student-level factors within a multilevel framework. Specifically, many existing studies have relied primarily on single-level analytical approaches, which may overlook the nested nature of educational data where students are clustered within classrooms taught by specific teachers (Marsh et al., 2012). This represents a notable research gap, as it may underemphasize how macro-level teacher characteristics cross-levelly associate with micro-level student outcomes. Interpersonal relationships and psychological mechanisms are increasingly recognized as critical determinants of academic performance. Therefore, this study aims to examine how teacher–student relationships, teaching innovation, and teacher professional identity influence academic performance, as well as the mediating role of student self-efficacy. This research contributes to the literature by providing a multilevel perspective on post-pandemic education.

## 2 Literature review

### 2.1 Teacher professional identity, teacher-student relationship, and academic performance

A good learning environment can meet students' learning needs, including good teaching and learning in the classroom environment, the absence of disciplinary problems in the classroom, and teaching and learning practices that can improve students' mental health, and self-efficacy, learning beliefs and academic performance (Schweisfurth, 2015; Blazar and Kraft, 2017; Talsma et al., 2021). Schweisfurth (2015) further argued that academic performance can be achieved in a friendly school environment, and that the teacher–student relationship is a critical factor in creating a student-friendly school environment. When creating a good learning atmosphere, teachers' most potent tool is creating positive relationships with their students (Boynton and Boynton, 2005). Jones and Kessler (2020) pointed out that teachers' critical roles in supporting students' learning and their social and emotional health have been widely discussed. Well-loved and inspiring teachers are popular in mass media and can build learning environments and curricula that meet children's needs. Such information has a significant impact on teachers' self-concepts and identities (Jones and Kessler, 2020).

From the perspective of identity theory (Brenner et al., 2018), people develop and retain their teacher identity. Hanna et al. (2019) evaluated teacher identity across 6 domains: self-image (view of the self as a teacher), motivation (reason for teaching), commitment (dedication to the profession), self-efficacy (ability to carry out activities as a teacher), task perception (understanding of a teacher's tasks), and job satisfaction (satisfaction with teaching). Jones and Kessler (2020) pointed out that teacher identity is fluid and socially constructed. That is, teacher identity is influenced by power and knowledge discourse (conveying what teachers should and should not do), and is reflective and forward-looking by virtue of emotions (conveying input) and dynamism (in constant negotiation with the above factors). Zhang et al. (2016) defined teachers' professional identity as a dynamic, multi-dimensional psychological and behavioral orientation toward the teaching profession, based on related value judgments and emotional experiences. Establishing good teacher–student relationships is the foundation of teacher happiness and an essential source of job satisfaction. It provides meaning and purpose, is connected to teachers' self-esteem, and provides overall trusted support for the profession (Lavy and Naama-Ghanayim, 2020). Distinct from teacher motivation, commitment, and self-efficacy, teacher professional identity represents a core self-conceptualization of one's professional role rather than task-specific or institutional attachments (Zhang et al., 2016). Within a multilevel framework, this robust professional self-concept may serve as a stable environmental input that enhances the classroom climate, thereby potentially fostering student-level cognitive and academic outcomes (Klassen and Tze, 2014). Therefore, teachers consider their status meaningful and work harder, contributing to their students' academic performance (Hanna et al., 2019; Lavy and Naama-Ghanayim, 2020).

The nature of teacher–student relationships in the school environment has attracted the attention of education scholars (Baafi, 2020). A favorable school environment refers to school settings and student-teacher interactions that increase student expectations, levels of self-confidence, and motivation for self-directed learning (Baafi, 2020). It includes a supportive learning atmosphere, a positive classroom teaching environment, and the availability of physical and mental health support from teachers and fellow students. Positive teacher–student relationships are related to higher academic performance among students, prosocial behavior, school adaptation, and academic success (Pianta and Stuhlman, 2004). Therefore, the first step in helping students become more motivated and engaged to achieve academic success is to establish and maintain positive teacher–student relationships. When a teacher fosters a welcoming and friendly environment and considers students' needs, students will effectively perform tasks that they deem necessary or interesting (Maulana et al., 2013).

Research on the role of teacher–student relationships in academic achievement has primarily been based on Self-Determination Theory (SDT), which posits that teacher–student interactions and student participation jointly determine students' academic achievement and can help them achieve ideal student performance (Jönsson, 2018). A positive teacher–student relationship improves the classroom and environment, thereby stimulating more motivation, which is essential for maintaining youth interest and learning engagement (Maulana et al., 2013) and promoting a strong interest in academic participation (Lee et al., 2019). Bartholomew et al. (2018) found that students were more likely to seek help from their teachers if they believed that their teachers were supportive and capable. Canales and Maldonado (2018) found that students who had positive relationships with their teachers and those who received support from their teachers tended to have better academic performance (Longobardi et al., 2016). Moreover, student self-efficacy emerged as a partial mediator in the relationship between student-teacher interaction and academic achievement (Gebresilase et al., 2025). In summary, this study explored the impact of teacher–student relationships and teacher professional identity on student performance, and whether such an impact is caused by student self-efficacy.

### 2.2 Teaching innovation and academic performance

Teaching innovation refers to teachers introducing new (self-developed or developed by others) concepts, strategies, and methods in the teaching process (Nurutdinova et al., 2016). Lee (2011) argues that teaching innovation involves the application of new teaching concepts, methods, or tools developed by oneself or others. It requires teachers to have an open mind and use communication skills such as reflection, questioning, deconstruction, and reconstruction to guide students to learn correctly and develop critical thinking and creative abilities. Novel and creative methods, strategies, and processes are used to make teaching lively and varied, thereby improving students' learning interest and effectiveness (Nurutdinova et al., 2016). Creative and innovative teaching methods can help students grasp and become more interested in specific concepts and create long-term memories or associations with concepts, thereby improving the teaching effect (Chang and Hsiao, 2016; Blegur et al., 2019). There is a positive correlation between creative and innovative teaching methods and students' academic performance (Narayanan, 2017; Suárez et al., 2025). Therefore, students need teachers to use innovative teaching methods that involve more active involvement (Sánchez Prieto et al., 2021). Consequently, this study explores the impact of teacher-teaching innovation on student performance and the mediating role of student self-efficacy in teaching innovation and student performance.

### 2.3 Self-efficacy and academic performance

Bandura (1977) proposed the concept of self-efficacy to explore human achievement behaviors from a social cognition perspective. He argues that “self-efficacy”” is the individual's ability to perceive and judge whether they can complete their work in a specific situation. Social cognitive theory (SCT) was developed based on the notion that learning is affected by individual cognitive, behavioral, and environmental factors. Self-efficacy is a critical element in the SCT, as it affects students' motivation and learning (Bandura, 1997; Pajares, 2006). Bandura (1997) and Pintrich and Schunk (1996) defined students' self-efficacy as their belief that they can use their abilities to meet their expectations of behavioral tasks and spontaneously construct and understand their perceptions of learning activities. Therefore, in the learning process, students first measure their level of learning, then evaluate their learning effectiveness, and finally decide on their learning behavior.

Students with high levels of self-efficacy can better overcome institutional obstacles (Bandura, 2011) as they strongly believe in their ability to succeed in a specific situation (Samfira and Palos, 2021), which may be necessary to adapt to remote learning. Oreshkina and Greenberg (2011) argued that teachers' beliefs about their potential help students learn. Conversely, underachieving students with low self-efficacy may believe that their efforts have been futile, negatively impacting their academic performance (Klassen, 2010). Many studies have reported that students' self-efficacy can directly predict academic performance (Doménech-Betoret et al., 2017; Miao et al., 2025; Moraga-Pumarino et al., 2025; Talsma et al., 2021; Xu and Qi, 2019; Yokoyama, 2019). In conclusion, this study attempts to integrate Social Cognitive Theory (SCT; Bandura, 1986) and Self-Determination Theory (SDT; Deci and Ryan, 2000) to construct a complementary, cross-level framework explaining how nested educational factors may associate with academic success.

Conceptually, these two theories could complement each other by bridging environmental conditions with cognitive mechanisms. In our proposed model, teacher professional identity and teaching innovation are grounded in SCT as potential environmental inputs (Klassen and Tze, 2014), while the teacher–student relationship is grounded in SDT as the relational context that may fulfill students' basic psychological need for relatedness (Deci and Ryan, 2000). While SDT helps clarify the motivational and relational conditions that support student growth, SCT explains the subsequent cognitive processing of these external resources.

Together, these frameworks map onto a sequential, cross-level pathway. Specifically, teacher-level environmental and relational factors (Level 2: identity, innovation, and relationships) are conceptualized to jointly support students' personal cognitive resources. According to the tenets of SCT, this supportive classroom environment may foster student self-efficacy (Level 1 cognition), which could subsequently act as a proximal psychological mechanism supporting student academic performance (Level 1 behavior). By tentatively linking each variable to these integrated pathways, this study seeks to provide a more holistic perspective on current higher education dynamics.

## 3 Methodology

### 3.1 Research framework

**Figure 1:**
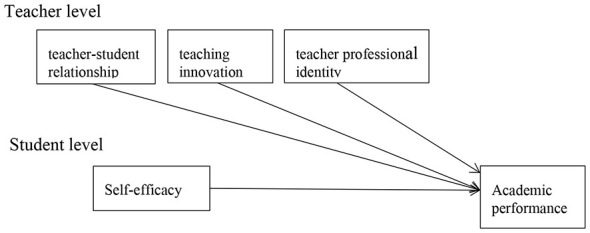
Research model.

SCT and SDT were used to explore the impact of teacher-related factors on students' academic performance (Figure 1), and HLM was used to validate the research model. The research model comprises two levels: teacher and student. The first includes teacher–student relationships, teaching innovation, and teacher professional identity variables, whereas the second includes self-efficacy and academic performance variables.

### 3.2 Procedure and participants

This study aimed to explore the impact of teacher factors on students' academic performance by surveying teachers and students at colleges in Hainan Province, China. The subjects of this study are third-year college students, because they had had online classes during the pandemic the previous year. Additionally, utilizing a convenience sampling method, the researchers contacted teachers via email in September 2023. This correspondence outlined the study's purpose and scope, requested their participation in the survey, and invited them to forward the online survey invitation to students under their supervision who were willing to participate. Each participating teacher completed the teacher questionnaire, whereas the students supervised by that teacher completed the student questionnaire. Student responses were subsequently matched with the corresponding teacher's questionnaire, with teachers serving as the Level 2 units and students as the Level 1 units in the hierarchical linear modeling (HLM) analyses. Before completing the online questionnaires, all participants (teachers and students) were informed about the purpose and procedures of the study. Participants were assured that participation was voluntary, that their responses would remain anonymous, and that they could withdraw from the study at any time without penalty. The study was approved by the Research Ethics Committee of Hainan Vocational University.

In this study, 40 teachers from 10 colleges in Hainan, each of whom taught 10–15 students, were surveyed. A total of 527 student questionnaires were recovered; 109 students were excluded because of invalid questionnaires (i.e., missing answers, selecting the same answer for nearly every question, or answering time being very short or very long), resulting in a final valid sample size of 40 teachers and 418 students. Although some studies recommended at least 50 Level 2 units for more accurate estimation of standard errors, previous simulation studies also indicated that multilevel modeling can still be performed when the number of Level 2 units is greater than 30 (Maas and Hox, 2005).

Teachers and students were categorized into different levels, and students were nested under teachers. Therefore, scholars suggest that a multilevel perspective is required to fully understand this context (Raudenbush and Bryk, 2002), as disregarding nested data structures may lead to aggregation bias, mis-estimated standard errors, and heterogeneity of regression (Raudenbush and Bryk, 2002). Thus, this study used HLM to analyze teacher-related factors and students' academic performance.

### 3.3 Instruments

#### 3.3.1 Teacher-student relationship, teaching innovation and professional identity

Teacher-level constructs were measured using data from only 40 teachers (Level 2). Therefore, the confirmatory factor analysis (CFA) results should be interpreted with caution. Previous methodological studies have shown that covariance-based CFA fit indices, particularly χ^2^, RMSEA, CFI, and TLI, are highly sensitive to small sample sizes and may underestimate model fit when the sample is limited (Boomsma, 1982; Marsh et al., 2004; Smid and Rosseel, 2020; Kline, 2023). Accordingly, in addition to reporting the global fit indices, this study primarily evaluated construct validity using standardized factor loadings, composite reliability (CR), and average variance extracted (AVE), which are recommended indicators of convergent validity (Fornell and Larcker, 1981; Hair et al., 2019).

Teacher–student relationships and teaching innovation were measured using Huang's (2006) Science Teacher School Environment Questionnaire. The survey included five items on the teacher–student relationship scored on a 5-point Likert scale. According to the reliability analysis, Cronbach's α was 0.890. CFA results indicated standardized factor loadings ranging from 0.599 to 0.895, CR = 0.893 (>0.70), and AVE = 0.629 (>0.50), supporting satisfactory convergent validity. Regarding model fit, SRMR = 0.019, χ^2^/df = 0.417, RMSEA = 0.000, GFI = 0.981, AGFI = 0.942, NFI = 0.982, IFI = 1.027, and CFI = 1.000 all exceeded the recommended criterion of 0.90 (Hu and Bentler, 1999), whereas PGFI = 0.327 and PNFI = 0.491 were below the recommended value of 0.50. Overall, most fit indices indicated an acceptable model fit.

The survey included five items measuring teaching innovation using a 5-point Likert scale. Cronbach's α was 0.886. CFA results showed standardized factor loadings ranging from 0.629 to 0.930, CR = 0.878 (>0.70), and AVE = 0.596 (>0.50), supporting satisfactory convergent validity. However, several global fit indices did not reach the recommended thresholds (SRMR = 0.100, RMSEA = 0.320, χ^2^/df = 4.991, GFI = 0.796, NFI = 0.808, IFI = 0.804, CFI = 0.833, PGFI = 0.265, and PNFI = 0.404). Given the limited Level-2 sample size (*n* = 40), these fit indices may reflect the statistical limitations associated with covariance-based CFA rather than inadequate psychometric quality of the scale (Boomsma, 1982; Marsh et al., 2004; Kline, 2023). Therefore, greater emphasis was placed on the convergent validity evidence, including factor loadings, CR, and AVE, all of which satisfied the recommended criteria. Nevertheless, the global fit indices should be interpreted cautiously, and future studies with larger teacher samples are recommended to further validate the measurement model.

Teacher professional identity was measured using the Teacher Professional Identity Scale developed by Zhang et al. (2016), consisting of 15 items rated on a 5-point Likert scale. Cronbach's α was 0.957. CFA results showed standardized factor loadings ranging from 0.504 to 0.889, CR = 0.958 (>0.70), and AVE = 0.609 (>0.50), indicating satisfactory convergent validity. The model fit indices were SRMR = 0.078, RMSEA = 0.224, χ^2^/df = 2.959, GFI = 0.593, NFI = 0.634, IFI = 0.723, CFI = 0.717, PGFI = 0.445, and PNFI = 0.543, with only SRMR and χ^2^/df meeting the recommended criteria. Similar to the teaching innovation scale, these global fit indices should be interpreted cautiously because all teacher-level constructs were estimated from the same limited Level-2 sample (*n* = 40). Considering the satisfactory standardized factor loadings, CR, and AVE, the scale demonstrated acceptable convergent validity despite the suboptimal global fit indices.

#### 3.3.2 Students' self-efficacy

Students' self-efficacy was measured using the self-efficacy scale of Scholz et al. (2002), which consists of 10 items (e.g., “I am certain that I can accomplish my goals”) scored on a 5-point Likert scale. Cronbach's alpha for the scale was 0.887. The CFA results, with factor loadings between 0.469 and 0.817, CR = 0.891, and AVE = 0.455, indicate that the items have good component reliability and construct validity. The values of SRMR = 0.047, χ^2^/df = 4.790, GFI = 0.926, AGFI = 0.884, PGFI = 0.589, NFI = 0.909, IFI = 0.927, CFI = 0.926, PNFI = 0.707, and RMSEA = 0.095 all indicate a good fit.

#### 3.3.3 Students' academic performance

Students' academic performance, measured on a scale of 0–100 points, was based on the average total score for all courses from February 2023 to June 2023, the entire semester after the students returned to school.

### 3.4 Data analysis

The data analysis for this study was carried out in four sequential steps: First, Confirmatory Factor Analysis (CFA) was performed to evaluate the validity and reliability of the measurement scales. Second, Descriptive Statistics and Preliminary Analysis were conducted to calculate the means and standard deviations (SD) for all study variables. Third, Common Method Variance (CMV) was assessed. Because data were collected via both student and teacher questionnaires, Harman's single-factor test was employed to ensure that CMV did not pose a significant threat to the validity of the findings. Fourth, Multilevel Model Analysis (Hierarchical Linear Modeling, HLM) was utilized to account for the nested structure of the data. Before testing the hypotheses, ICC(1) and ICC(2) values were calculated to justify the necessity of multilevel modeling. The analysis was then executed across two distinct levels: Level 1 (Student Level): self-efficacy and Academic performance were modeled as individual-level variables. Level 2 (Teacher Level): teacher–student relationship, Teaching innovation, and Teacher professional identity were specified as organizational-level predictors.

## 4 Research results

In all, 40 teachers and 418 students at 10 schools completed the questionnaires. Among the teachers, 17 were male (42.5%) and 23 female (57.5%), while of the 418 students, 150 were male (35.9%) and 268 female (64.1%). Regarding teachers' scores for each variable, the mean scores for teacher–student relationships, teaching innovation, and teacher professional identity were 2.06, 3.92, and 3.82, respectively. For students, the mean score for academic performance and self-efficacy was 80.86 and 3.62, respectively. The normality test results indicate that, for teachers, the skewness of the teacher–student relationship, teaching innovation, and teacher professional identity falls between −0.116 and −1.262, with an absolute value of less than 2. The kurtosis is between −1.277 and 1.855, also with an absolute value less than 2. For students, the skewness of self-efficacy and academic performance were −0.271 and 0.201, respectively, while the kurtosis was 0.222 and 0.830. Both the skewness and kurtosis values were less than 2, indicating that the sample conformed sufficiently to a normal distribution (Curran et al., 1996). The descriptive statistics for each variable are presented in Table 1.

**Table 1:** Descriptive statistics of variables.

Variable	*N*	Mean	SD	Minimum	Maximum	Skewness	Kurtosis
Teacher level							
Teacher-student relationship	40	2.06	0.58	1.20	3.00	−1.262	1.855
Teaching innovation	40	3.92	0.49	3.00	4.60	−0.446	−0.953
Teacher professional identity	40	3.82	0.67	3.00	4.93	−0.116	−1.277
Student level							
Self-efficacy	418	3.62	0.54	1.40	5.00	0.201	0.830
Academic performance	418	80.86	11.81	24.00	100.00	−0.271	0.222

Regarding the gender difference analysis in self-efficacy and academic performance, no significant difference was found in self-efficacy between male (*M* = 3.69) and female students (*M* = 3.58; *t* = 1.948, *p* > 0.05). However, a significant gender difference emerged in academic performance (*t* = 3.85, *p* < 0.001), with male students scoring significantly higher (*M* = 83.78) than female students (*M* = 79.22).

### 4.1 Common method variance

Several procedural and statistical remedies were adopted to reduce common method variance (CMV). First, participants were assured of anonymity and confidentiality. Second, this study employed a multilevel and multi-source design, in which student-level variables were collected from students and teacher-level variables were collected from teachers, thereby reducing common source bias.

Third, Harman's single-factor test showed that the first unrotated factor explained less than 40% of the total variance, indicating that CMV was not a serious concern. In addition, the aggregation of teacher-level variables was supported by acceptable intraclass correlation coefficients [ICC(1) = 0.073; ICC(2) = 0.766], indicating adequate between-group variance and reliability for hierarchical linear modeling (Bliese, 2000; James et al., 1984). Therefore, CMV was unlikely to substantially affect the findings of this study.

### 4.2 Multilevel model analysis

When conducting HLM analysis, the cross-level effect must be examined using an unconstrained (null) model before further stochastic and intercept associative analyses are performed. When the intraclass correlation coefficient (ICC) value is greater than 0.059 (Cohen, 1988) and ICC2 is greater than 0.50 (James et al., 1993), the between-group variation is significantly different, and a cross-level statistical analysis must be considered.

### 4.3 Unconstrained (null) model

In analyzing the unconstrained (null) model of academic performance, the component value of between-group variation was 10.233, which was significant (χ^2^ = 37.929, df = 9), and that of within-group variance was 130.387, which satisfied the requirement for between- and within-group variance of dependent variables in HLM analysis (Gavin and Hofmann, 2002). The ICC was 10.233/(10.233 + 130.387) = 0.073, ICC2 = 0.766. Only 7.3% of the variation came from the school level, with the majority coming from students (93.9%). As ICC = 0.073 > 0.059 and ICC2 = 0.766 > 0.5, cross-level statistical analysis was performed.

### 4.4 Stochastic predictive model at the student level

The impact of self-efficacy on academic performance was analyzed using a random parameter regression model as follows:

Level-1 model


$$\begin{array}{l} \text{Academic performanc}{\text{e}}_{\text{ij}}=\text{β}0_{\text{j}}+\text{β}1_{\text{j}}*\left(\text{self}-\text{efficacy}\right)+\text{rij} \end{array}$$

**Figure 2:**
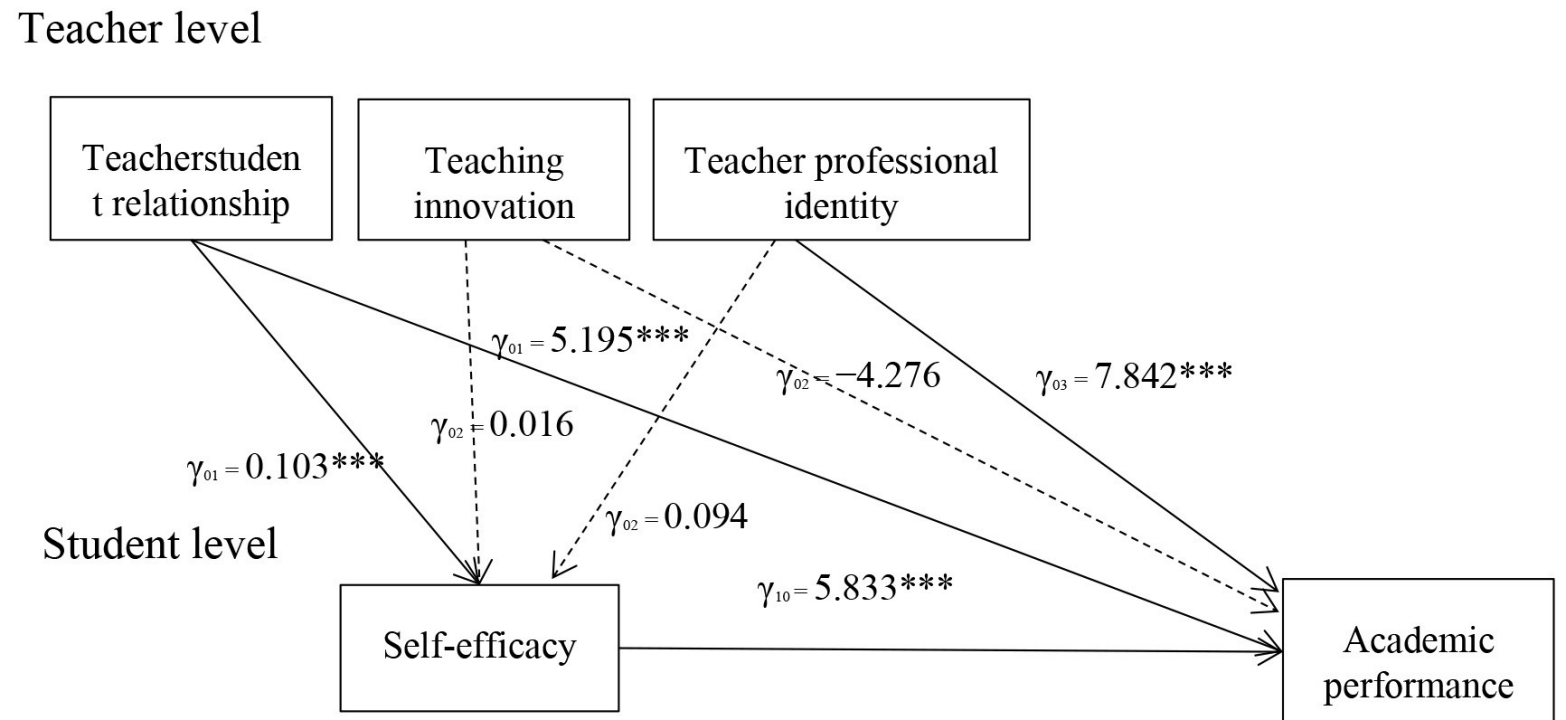
HLM estimation results. ***p < 0.001 The dotted line means that it is not significant.

The results demonstrate that γ_10_ is significant (γ_10_ = 5.833, *t* = 7.215, *p* < 0.001), indicating that self-efficacy has a significantly positive impact on academic performance (Table 2 and Figure 2). The higher the students' self-efficacy, the better their academic performance, and vice versa.

**Table 2:** Summary of HLM analysis results.

Model (dependent variable)	Model 1 (academic performance)		Model 2 (academic performance)		Model 3 (self-efficacy)		Model 4 (academic performance)	
Student level	Coefficient	*t*	Coefficient	*t*	Coefficient	*t*	Coefficient	*t*
γ00	59.817	17.951^***^	56.987	5.807^***^	2.982	25.918^***^	42.080	5.652^***^
Self-efficacy γ10	5.833	7.215^***^					5.688	6.658^***^
School level								
Teacher-student relationship γ01			5.195	4.191^**^	0.103	7.232^***^	4.369	3.797^*^
Teaching innovation γ02			−4.276	−2.186	0.016	0.456	−5.003	−2.749^*^
Teacher professional identity γ03			7.842	2.501^*^	0.094	2.175	7.551	2.765^*^
Random effect	Variation	χ^2^	Variation	χ^2^	Variation	χ^2^	Variation	χ^2^
*u*00	31.331	8.675	5.923	17.348^**^	0.001	1.903	17.463	7.662
*u*10	0.6012	6.680					0.330	6.617
γij	121.490		130.373		0.289		121.552	

^*^p < 0.05, ^**^p < 0.01, ^***^p < 0.001.

### 4.5 Stochastic predictive model at the learning environment level and student level

The teacher level includes teacher–student relationships, teaching innovation, and teacher professional identity. The stochastic predictive model can be expressed as follows:

Level-1 model


$$\begin{array}{l} \text{Academic performanc}{\text{e}}_{\text{ij}}=\text{β}0_{\text{j}}+\text{β}1_{\text{j}}*\left(\text{self}-\text{efficacy}\right)+{\text{r}}_{\text{ij}} \end{array}$$

Level-2 model


$$\begin{array}{r} \text{β}0_{\text{j}}=\gamma_{00}+\gamma_{01}*\left(\text{teacher}-\text{studentrelationship}\right)\\ +\gamma_{02}*\left(\text{teachinginnovation}\right)\\ +\gamma_{03}*\left(\text{teacherprofessionalidentity}\right)+{\text{u}}_{0\text{j}} \end{array}$$

Regarding the teacher and student levels, Model 2 in Table 2 and Figure 2 indicates that the teacher–student relationship and teacher professional identity had a positive and significant association with students' academic performance (γ_01_ = 5.195, *t* = 4.191, *p* = 0.007; γ_03_ = 7.842, *t* = 2.501, *p* = 0.046). Teaching innovation was not significantly associated with academic performance (γ_02_ = −4.276, *t* = −2.186, *p* = 0.071). These results indicate that the better the teacher–student relationship, the better students' academic performance. Teachers' professional identity with their own identities can also improve their students' academic performance. However, teachers' teaching innovations had no significant impact on students' academic performance.

### 4.6 The mediating effect model of student self-efficacy on teacher factors and student academic performance

To test the mediating effect of student self-efficacy, we first analyzed the impact of school environment factors, such as teacher–student relationships, teaching innovation, and teacher professional identity, on student self-efficacy. The intercept prediction model was then used to analyze the mediating effect of student self-efficacy on teacher–student relationships, teaching innovation, teacher professional identity, and academic performance.


$$\begin{array}{l} \text{Academic performanc}{\text{e}}_{\text{ij}}=\text{β}0_{\text{j}}+\text{β}1_{\text{j}}*\left(\text{self}-\text{efficacy}\right)+{\text{r}}_{\text{ij}} \end{array}$$

Level-2 model


$$\begin{array}{r} \text{β}0_{\text{j}}=\gamma_{00}+\gamma_{01}*\left(\text{teacher}-\text{studentrelationship}\right)\\ +\gamma_{02}*\left(\text{teachinginnovation}\right)\\ +\gamma_{03}*\left(\text{teacherprofessionalidentity}\right)+{\text{u}}_{0\text{j}} \end{array}$$

The results of Model 3 for the effects of teacher–student relationship, teaching innovation, and teacher professional identity on student self-efficacy demonstrate that only the teacher–student relationship has a positive and significant impact on student self-efficacy (γ_01_ = 0.103, *t* = 7.232, *p* = 0.000); neither teaching innovation nor teacher professional identity has a significant impact on students' self-efficacy (γ_02_ = 0.016, *t* = 0.456, *p* = 0.664; γ_03_ = 0.094, *t* = 2.175, *p* = 0.072). The mediation effect test of Model 4 then found that teacher–student relationship has a positive and significant impact on student's academic performance (γ_01_ = 4.369, *t* = 3.797, *p* = 0.012); students' self-efficacy also has a positive and significant impact on academic performance (γ_10_ = 5.688, *t* = 6.658, *p* = 0.000), and Following Preacher (2012) Monte Carlo method for multilevel mediation analysis, the indirect effect of teacher–student relationship on students' academic performance through students' self-efficacy was significant [ab = 0.586, 95% CI = (0.369,0.839)], as the confidence interval did not include zero. indicating that it has a positive mediating effect between the teacher–student relationship and academic performance (Table 2 and Figure 2). A good teacher–student relationship can thus enhance student self-efficacy, thereby enhancing academic performance.

The results indicate that teacher–student relationships are significantly and positively associated with academic performance. Teacher professional identity also shows a positive association. In contrast, teaching innovation does not have a significant impact. At the student level, self-efficacy is significantly associated with academic performance and partially mediates the relationship between teacher–student relationships and academic outcomes.

## 5 Discussion

The findings indicate that teacher–student relationships and teacher professional identity are positively associated with academic performance, whereas teaching innovation is not significantly associated with academic performance. Moreover, students' self-efficacy was positively associated with academic performance. Although the teacher–student relationship was positively associated with students' academic performance, its mean value was 2.06, indicating that teachers generally believed that the relationship was poor. This rating could reflect the fewer interactions between teachers and students during online classes, which may have limited teachers' opportunities to provide guidance, assistance, and feedback to underachieving students (Bakken et al., 2020). Furthermore, students were confused about what their teachers required. These reasons may have made it difficult to improve teacher–student relationships after schools reopened (Mælan et al., 2021). Another reason could be non-teaching factors, such as teachers taking care of vulnerable family members, homeschooling their children, or managing their mental health (Kim and Asbury, 2020). Furthermore, although college students have returned to school, many elementary schools, kindergartens, and companies have not reopened. Consequently, teachers may not have been able to focus entirely on their students. Thus, further efforts are required to improve or reestablish teacher–student relationships to better understand their association with students' academic performance.

Teaching innovation was not significantly associated with students' academic performance. This may be because teachers and students had to adapt quickly to distance learning when their schools were closed (Carrillo and Flores, 2020). Moreover, teachers had to design online teaching courses and familiarize themselves with new teaching systems. However, after schools reopened, teachers had to re-establish face-to-face classes, which may have placed additional pressure on them (Kim and Asbury, 2020). Teachers constantly learned and used new teaching methods as schools closed and reopened. However, this does not necessarily indicate innovation. Thus, teaching innovation was not significantly associated with students' academic performance.

However, the association of instructional innovation with students' academic performance was not statistically significant (γ02 = −4.276, *p* = 0.071). This finding may be partly attributable to the relatively small Level 2 sample size (*n* = 40) in the present study. Previous multilevel modeling studies have suggested that insufficient Level 2 sample sizes may reduce statistical power and inflate *p*-values, thereby making it more difficult to detect significant group-level effects (Maas and Hox, 2005; Raudenbush and Bryk, 2002). Accordingly, the marginally non-significant finding in this study should be interpreted with caution, as the association between instructional innovation and academic performance may have been underestimated due to the limited Level 2 sample size. Future studies with larger group-level samples are recommended to further examine the robustness of this cross-level association.

Teacher professional identity in Chinese colleges was positively associated with students' academic performance. Teachers believe that being a teacher is a rewarding career that comes with a sense of accomplishment, self-esteem, and trust (Lavy and Naama-Ghanayim, 2020), which is associated with greater dedication to their profession (Brenner et al., 2018; Hanna et al., 2019). Hence, teachers with relatively high levels of professional identity also tended to report higher levels of attention to their students' learning and lives. Correspondingly, higher teacher professional identity was positively associated with better academic performance among college students.

Chinese college students' self-efficacy was positively associated with academic performance, a finding consistent with previous studies (Talsma et al., 2021). Students displayed better academic performance despite the pandemic when they believed they were working hard and solving learning-related problems. This also indicates that students' self-efficacy remained positively associated with academic performance even after returning to school.

This study highlights the importance of interpersonal relationships and psychological mechanisms in post-pandemic education. Teacher–student relationships were directly and indirectly associated with academic performance through self-efficacy, consistent with social cognitive theory and self-determination theory. Teacher professional identity was also positively associated with academic performance, suggesting that relational alignment may play an important role. In contrast, teaching innovation was not significantly associated with academic performance, indicating that relational stability may be more closely associated with academic performance than instructional novelty in the post-pandemic context.

## 6 Conclusion

This study examined the relationships among college teacher factors and students' academic performance after returning to school over an entire semester in Hainan, China. College teacher–student relationships, teacher professional identity, and students' self-efficacy were positively associated with students' academic performance. In contrast, teachers' teaching innovation was not significantly associated with students' academic performance. After college students returned to school, establishing positive teacher–student relationships may help address the deterioration of teacher–student relationships experienced during online classes. Furthermore, teachers should pay attention to students' self-efficacy, whether in online or offline learning environments, because students' self-efficacy was positively associated with their academic performance.

## 7 Recommendations

These findings suggest that when college students return to school for face-to-face learning, teachers should quickly establish excellent teacher–student relationships to compensate for their reduced ability to provide care, immediate feedback, and assistance to students during online classes. They should also attempt to improve the teacher–student relationships when teaching online.

Schools must also take care of teachers and help them reduce family-related burdens to ameliorate the negative impacts of non-teaching factors. Finally, schools should enhance teachers' professional identity. Teachers with a high degree of identification with their profession put more effort into their work, ultimately boosting their academic performance. Although the study subjects were students from only Hainan, China, many schools worldwide are currently transitioning from online to offline courses or are combining both teaching methods; therefore, the results and suggestions of this study can serve as a reference for them.

## 8 Limitations

This study has several limitations. First, the study did not survey students during the pandemic; therefore, the findings may not fully reflect changes in teaching practices and students' learning experiences under pandemic-related educational conditions. Second, the present study could not fully explain why college teachers' teaching innovation had no significant impact on students' academic performance. One reason may be the relatively small Level-2 sample size (*N* = 40). Although multilevel modeling can be conducted with more than 30 Level-2 units (Maas and Hox, 2005), estimates—particularly standard errors and cross-level effects—may still exhibit instability when multiple Level-2 predictors are included simultaneously. Given the number of teacher-level predictors in our model, this sample size limitation might have reduced the statistical power at the teacher level, suggesting that care should be taken when interpreting the cross-level relationships. Third, this study adopted a cross-sectional design, which limits the ability to infer causal relationships among the variables. Fourth, the present study did not further examine potential gender differences in the proposed relationships. Fifth, the assessment of the teacher-student relationship relied exclusively on teachers' perspectives, which may introduce single-source bias. Because relationships are inherently dyadic, relying on a single informant may not fully capture the shared reality of the classroom interactions. Finally, the geographical scope of the sample was limited to Hainan Province, China, which may restrict the generalizability of the findings to other regions or educational contexts.

## Data Availability

The raw data supporting the conclusions of this article will be made available by the authors, without undue reservation.
